# Construction of ceRNA network based on RNA-seq for identifying prognostic lncRNA biomarkers in Perthes disease

**DOI:** 10.3389/fgene.2023.1105893

**Published:** 2023-05-26

**Authors:** Tianjiu Zhang, Xiaolin Hu, Song Yu, Chunyan Wei

**Affiliations:** ^1^ Guizhou Children’s Hospital, Department of Pediatric Surgery, Affiliated Hospital of Zunyi Medical University, Zunyi, Guizhou, China; ^2^ School of Public Health, Shanghai Jiao Tong University School of Medicine, Shanghai, China; ^3^ Department of Gynecoloay, Obstetrics and Gynecoloay Hospital of Fudan University, Shanchai, China

**Keywords:** Perthes disease, pathogenesis, ceRNA, RNA-seq, miRNA-seq

## Abstract

**Introduction:** Legg-Calvé-Perthes disease or Perthes disease is a condition that occurs in children aged 2 to 15 years, and is characterized by osteonecrosis of the femoral head, which results in physical limitations. Despite ongoing research, the pathogenesis and molecular mechanisms underlying the development of Perthes disease remain unclear. In order to obtain further insights, the expression patterns of long non-coding RNAs (lncRNAs), miRNAs, and mRNAs in a rabbit model of Perthes disease were analyzed in this study by transcriptome sequencing.

**Methods and results:** The results of RNA-seq analyses revealed that 77 lncRNAs, 239 miRNAs, and 1027 mRNAs were differentially expressed in the rabbit model. This finding suggested that multiple genetic pathways are involved in the development of Perthes disease. A weighted gene co-expression network analysis (WGCNA) network was subsequently constructed using the differentially expressed mRNAs (DEmRNAs), and network analysis revealed that the genes associated with angiogenesis and platelet activation were downregulated, which was consistent with the findings of Perthes disease. A competing endogenous RNA (ceRNA) network was additionally constructed using 29 differentially expressed lncRNAs (including HIF3A and LOC103350994), 28 differentially expressed miRNAs (including ocu-miR-574-5p and ocu-miR-324-3p), and 76 DEmRNAs (including ALOX12 and PTGER2).

**Disscusion:** The results obtained herein provide novel perspectives regarding the pathogenesis and molecular mechanisms underlying the development of Perthes disease. The findings of this study can pave the way for the development of effective therapeutic strategies for Perthes disease in future.

## 1 Introduction

Legg-Calvé-Perthes disease (Perthes disease) is one of the most commonly occurring necrotic diseases in children aged between 2 and 15 years, and is characterized by avascular necrosis of the femoral head (Herring et al., 2004; Rich and Schoenecker, 2013). The prognosis of Perthes disease is strongly associated with age, sex, socioeconomic deprivation, and severity of necrosis of the femoral head (Pavone et al., 2019). The common symptoms of Perthes disease include stiffness of the hip joint, pain, and limping, which decreases the quality of life of the affected children (Landin et al., 1987). The typical clinical methods for treating Perthes disease include the use of a hip-abduction plaster cast or brace immobilization, as well as proximal femoral varus osteotomy, which increases the inclusion of the hip joint (Beer et al., 2008; Nelitz et al., 2009; Rodríguez-Olivas et al., 2022).

The interruption of blood flow to the femoral head is considered to be a crucial factor in the development of Perthes disease (Atsumi et al., 2000). Recent studies investigating the genetic factors associated with Perthes disease have reported that the vascular endothelial growth factor (*VEGF*), Hypoxia Inducible Factor 1 (*HIF1*), and interleukin (IL)-6 are potential contributors to disease development (Kim, 2012; Yamaguchi et al., 2016). Previous studies have identified that *VEGF-a* is a key regulator of endochondral ossification, and studies in piglet (Kim et al., 2004) and rabbit (Li et al., 2008) models have demonstrated that the administration of *VEGF-a* restores endochondral ossification in epiphyseal cartilages. Perthes disease is also characterized by the occurrence of inflammation, which can affect bone remodeling. Interestingly, a previous study demonstrated that the levels of *IL-6* G174C/G597A are significantly lower in patients with Perthes disease (Srzentić et al., 2014). In the study by Ren et al., the administration of the anti-*IL-6* monoclonal antibody, tocilizumab, in a piglet model of Perthes disease significantly increased the bone volume and reduced the number of osteoclasts compared to those of the control group. Additionally, the number of synovial macrophages and vessels was significantly reduced in the group treated with tocilizumab (Srzentić et al., 2014). RNA-seq analysis of samples of femoral heads following fracture revealed that *FGF2*, *IGF1*, *SOX9*, *COL2A1*, and *FAM201A* could be involved in the osteonecrosis of the femoral head (Huang et al., 2018). It is believed that the pathogenesis of Perthes is mediated by a complex network of genes and gene-gene interactions, instead of a single gene (Pavone et al., 2019). We have previously demonstrated that gene-gene interactions being complexed in different disease (Wei et al., 2016; Hu et al., 2022). However, further studies are necessary for investigating the interactions and mechanism of regulation of these genes.

Next-generation sequencing technologies have enabled the comprehensive transcriptome-wide analysis of gene expression in various diseases in recent years. Previous studies have demonstrated that long non-coding RNAs (lncRNAs) act as competing endogenous RNAs (ceRNAs) by sponging miRNAs. This subsequently inhibits the exchange of miRNA response elements with mRNAs by increasing the competition for common miRNAs (Salmena et al., 2011). Growing evidence suggests that the abnormal expression of lncRNAs and miRNAs may be associated with the pathogenesis of Perthes disease. Using microarray data, Wang et al. developed a co-expression network of lncRNAs and mRNAs, and identified 13 lncRNAs that were significantly associated with the development of Perthes disease (Wang et al., 2022). Another study demonstrated that miR-214 promotes the viability of chondrocytes and reduces apoptosis by suppressing *Bax* (Zhu et al., 2019), while *miR-206* promotes cellular apoptosis in Perthes disease by suppressing the expression of *SOX9* (Luo et al., 2018a). Huang et al. identified 35 differentially expressed miRNAs (DEmiRNAs) in early Perthes disease by miRNA sequencing using plasma exosomes. Notably, the study revealed that the expression levels of *has-miR-3133*, *has-miR-4644*, *has-miR-150-5p*, and *hsa-miR-4693-3p* were significantly increased in the plasma exosomes, and that the *MAPK*, *Ras*, *cAMP*, and *PI3K-Akt* signaling pathways were involved in disease pathogenesis (Huang et al., 2022). The ceRNA hypothesis describes a comprehensive regulatory mechanism between coding and non-coding RNAs (Tay et al., 2014). Although several studies have investigated several mRNAs, lncRNAs, and miRNAs involved in the pathogenesis of Perthes disease, the role of comprehensive ceRNA remains poorly investigated to date.

The present study therefore aimed to construct a comprehensive ceRNA network of Perthes disease using a rabbit model for obtaining insights into the mechanisms and functions of genes involved in Perthes disease. To this end, a rabbit model of Perthes disease was established in this study, and the potential mechanisms underlying the pathogenesis of Perthes disease were investigated by whole transcriptome sequencing (total RNA-seq and miRNA-seq). Bioinformatics analysis revealed that a total of 1027 differentially expressed genes (DEGs), 77 differentially expressed lncRNAs (DElncRNAs), and 239 DEmiRNAs were involved in the pathogenesis of Perthes disease. The biological processes and pathways related to Perthes disease were subsequently identified by enrichment analysis. The DEGs were further clustered by weighted gene correlation network analysis (WGCNA), and two groups of DEGs were identified that were closely associated with known mechanisms of pathogenesis of Perthes disease, including angiogenesis and downregulation of platelet activation. An lncRNA-miRNA-mRNA regulatory network was finally constructed in this study. The findings of the present study provide novel insights into the pathogenesis of Perthes disease and can aid in identifying potential therapeutic targets and biomarkers of Perthes disease, which need to validated by further experiments.

## 2 Results

### 2.1 Generation of rabbit model of Perthes disease

**FIGURE 1:**
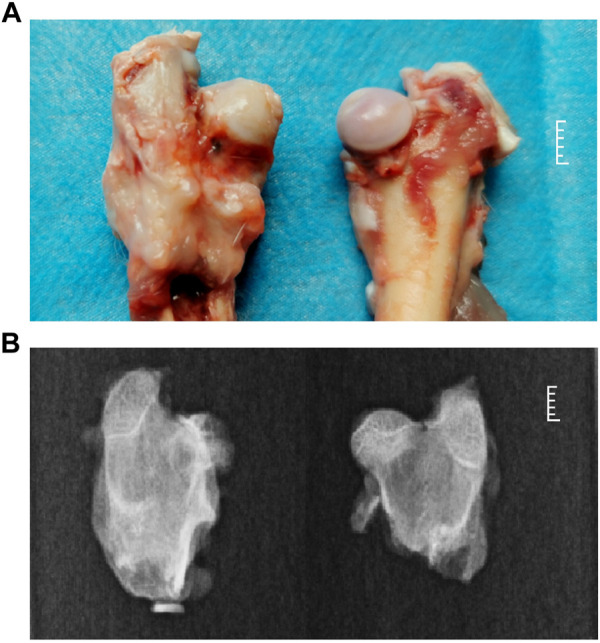
Phenotypic characteristics of the rabbit model of Perthes disease. **(A)** morphology of the femoral neck in the rabbit model of Perthes disease (left) and healthy control (right). **(B)** X-rays of the femoral neck of the rabbit model of Perthes disease (left) and healthy controls (right).

Five 8-week-old immature rabbits were selected for preparing the rabbit model of Perthes disease. The model was established based on morphological analysis and visualization with X-rays. In Perthes disease, the femoral head is characterized by the loss of spherical structure, pale color, thickened cartilage layer, and lack of gloss, as depicted in Figure 1A (left). X-rays of the femoral head revealed a heterogeneous bone density, local collapse, and loss of spherical structure (Figure 1B; left). These findings were consistent with previously reported observations in children with Perthes disease (Catterall et al., 1982; Madan et al., 2003; Trostel et al., 2003; Memiş et al., 2018).

### 2.2 Identification of DElncRNAs and DEmRNAs

**FIGURE 2:**
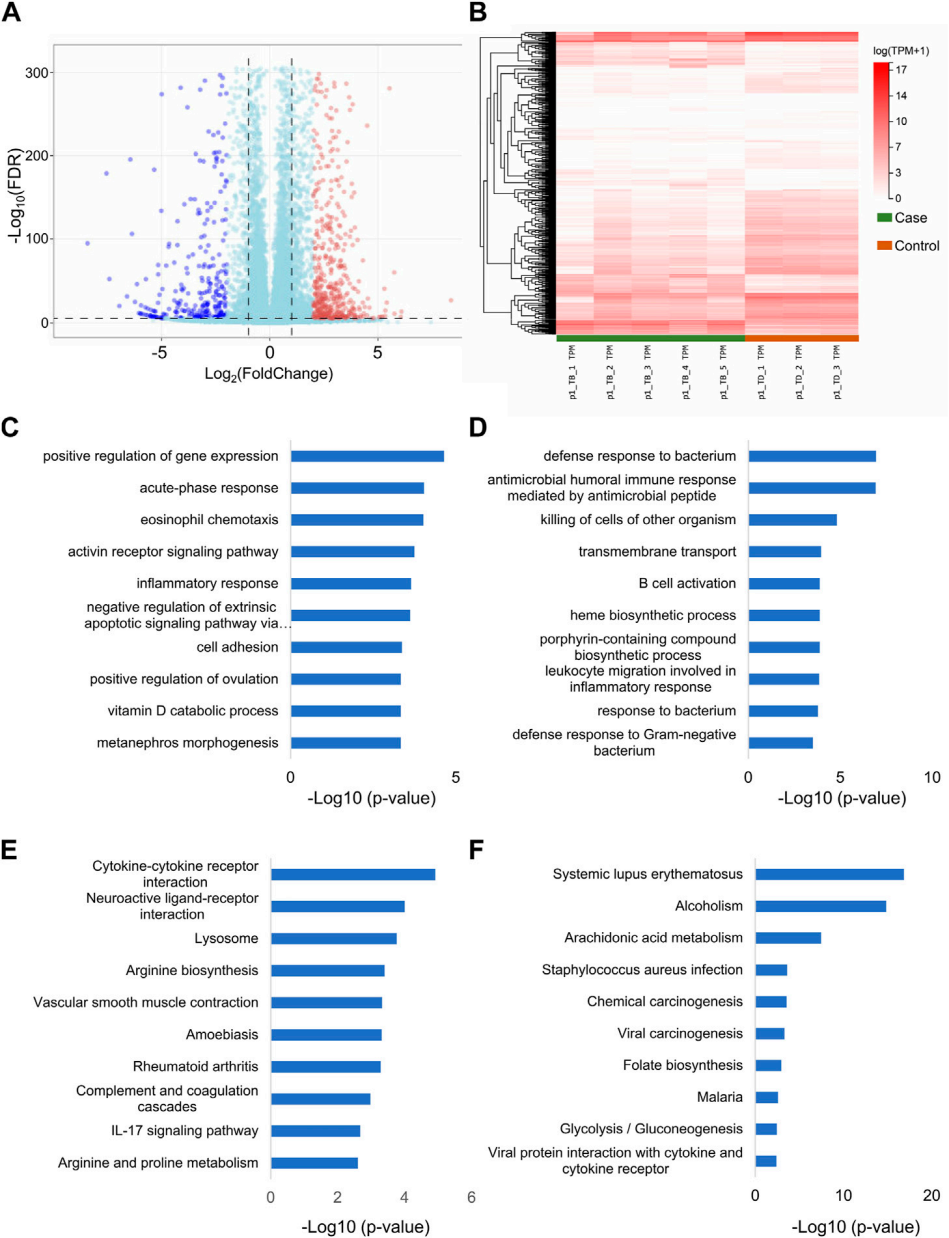
Differential expression of lncRNAs and mRNAs in Perthes disease. Heatmap depicting the **(A)** lncRNAs and **(B)** mRNAs that were differentially expressed between the model of Perthes disease and the control group. The gene expression levels were normalized as tanscripts pre kilobase million (TPM). The genes are depicted in different rows, while the columns indicate separate samples. TB and TD indicate samples corresponding to the Perthes disease model and control group, respectively. The results of Gene Ontology (GO) enrichment analysis of the **(C)** upregulated and **(D)** downregulated DEGs. Results of Kyoto Encyclopedia of Genes and Genomes (KEGG) pathway enrichment analysis of the **(E)** upregulated and **(F)** downregulated DEGs. The *x*-axis represents the log_10_ *p*-values, while the enriched terms are depicted along the *y*-axis. The sixth term in panel C is “negative regulation of extrinsic apoptotic signaling pathway via death domain receptors”.

The total RNA-seq data from 5 rabbits with Perthes disease and 3 control rabbits were analyzed for comprehensive identification of the genes associated with Perthes disease. The gene expression patterns of each sample were quantified and differential analysis was performed using the DESeq2 tool. A total of 1027 defferential expressed Genes (DEGs) (Supplementary Table S1) and 77 DElncRNAs (Supplementary Table S2) were identified, and genes with log_2_(fold change) > 2 or < −2 and FDR values < 1e-5 were considered to be significantly differentially expressed. Of these, 369 DEGs and 32 DElncRNAs were upregulated in the Perthes disease group, while 658 DEGs and 45 DElncRNAs were downregulated (Figures 2A, B). Notably, the DEGs identified in the rabbit model of Perthes disease in this study correlated with genes previously reported to be involved in inflammation [*IL-6* and *HIFa* (Zhang et al., 2015; Ren et al., 2021)] and angiogenesis [*PTGER2* (Trau et al., 2015) and *ALOX12* (Zheng et al., 2020)] in Perthes disease.

### 2.3 Determination of the biological functions of DEGs

Gene Ontology (GO) (Consortium, 2019) and Kyoto Encyclopedia of Genes and Genomes (KEGG) pathway (Kanehisa and Goto, 2000) enrichment analyses were performed using the clusterProfiler package in R (Yu et al., 2012) for obtaining a better understanding of the biological functions of the DEGs. The results of GO enrichment analyses revealed that the DEGs that were upregulated in Perthes disease were primarily enriched in gene expression regulation, acute phase response, eosinophil chemotaxis, activin receptor signaling, inflammatory response, and other terms in the biological process (BP) category of GO (Figure 2C; Supplementary Table S3). The downregulated DEGs were primarily enriched in defense response to bacterium, antimicrobial humoral immune response, B cell activation, and other terms, which indicated a weakened immune defense against bacterial infections (Figure 2D; Supplementary Table S4).

The results of KEGG pathway enrichment analysis revealed that the upregulated DEGs were most significantly enriched in the cytokine-cytokine receptor, neuroactive ligand, lysosome, and IL-17 signaling pathway, among others (Figure 2E; Supplementary Table S5), while the downregulated DEGs were primarily enriched in arachidonic acid metabolism, chemical, viral, and carcinogenesis pathways (Figure 2F; Supplementary Table S6).

As the DEGs were enriched in several GO and KEGG terms, it was necessary to analyze the genes with similar expression patterns for identifying the specific biological processes and pathways of the DEGs.

### 2.4 Construction of WGCNA network

**FIGURE 3:**
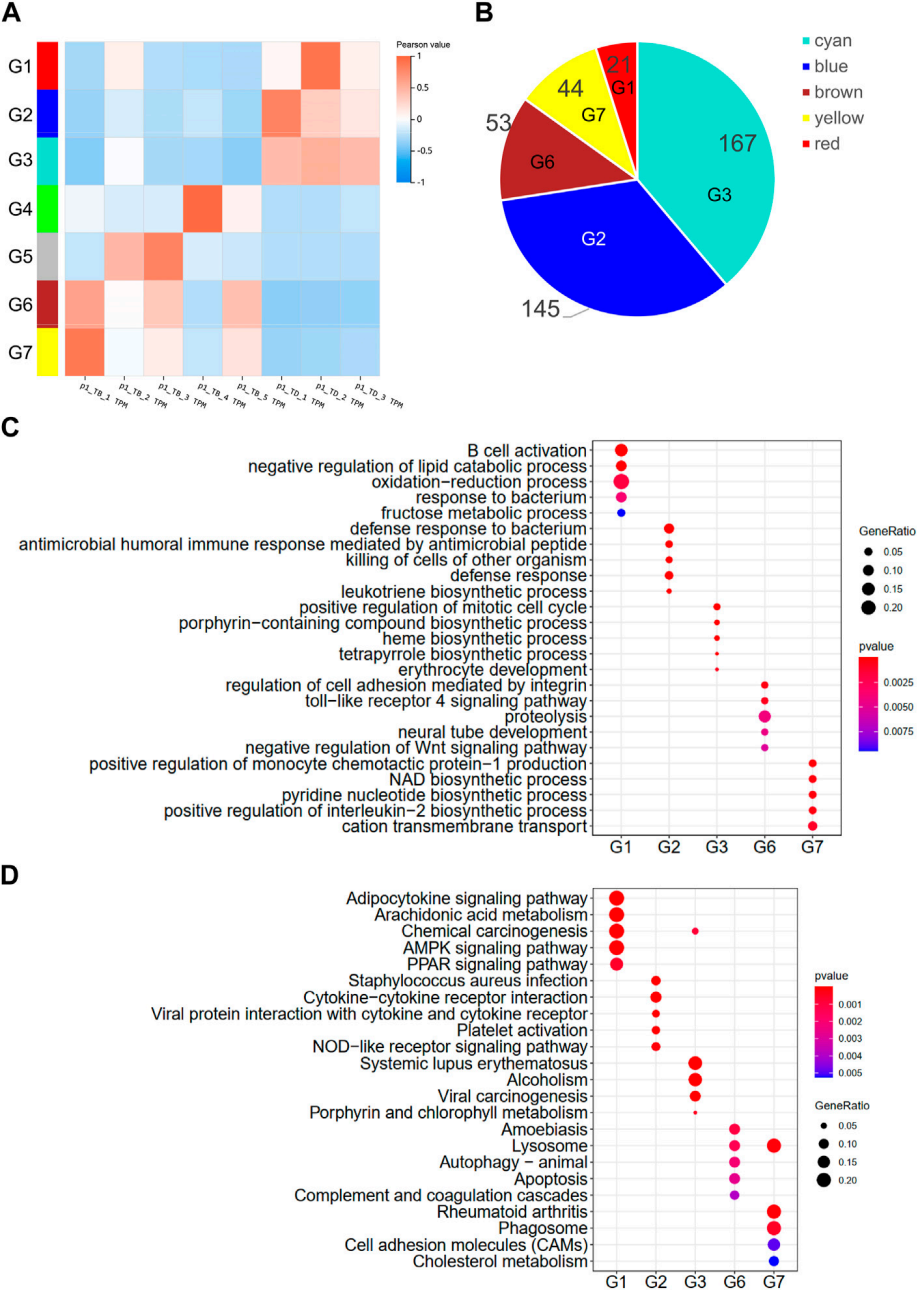
WGCNA of DEGs. **(A)** Hierarchical clustering heatmap depicting the co-expressed genes in different modules. The rows represent different samples, while the gene expression patterns of the different groups are depicted in separate columns. The color of each dot represents the Pearson value. **(B)** Number of DEGs in the different groups. Results of **(C)** GO and **(D)** KEGG enrichment analysis of the DEGs in the different groups.

WGCNA is a hierarchical clustering method that is used to analyze the gene expression patterns of multiple samples (Langfelder and Horvath, 2008). In this study, seven clusters of DEGs were identified by filtering the significant module scores (Figure 3A). The DEGs in the G1 (red), G2 (blue), and G3 (cyan) groups were highly expressed in all the control samples, while the DEGs in the G6 (brown) and G7 (yellow) groups were more highly expressed in the treatment groups that contained more than 4 disease samples. The G2 and G3 groups contained more than half of the identified DEGs (Figure 3B). The DEGs were subjected to GO and KEGG enrichment analyses for comparing the enriched biological processes. The results of enrichment analyses revealed that the DEGs in the different groups were enriched in completely different biological processes as depicted in Figure 3C; Supplementary Tables S7, S8. The DEGs in group G2 were primarily involved in proteolysis, *TLR4* pathway, and negative regulation of the Wnt pathway, which are known to be involved in the osteonecrosis of the femoral head (Jiang et al., 2014; Zhu et al., 2021). The DEGs in the group G1 were involved in *NAD* biosynthesis, pyridine nucleotide biosynthesis, positive regulation of *IL-2*, and cation transmembrane transport. The results of KEGG pathway enrichment analysis revealed that the DEGs in group G2 were enriched in apoptosis, lysosome, autophagy, and amoebiasis pathway, while the DEGs in group G1 were enriched in phagosome, cell adhesion molecules, cholesterol metabolism, and rheumatoid arthritis pathways (Figure 3D).

### 2.5 Identification of novel miRNA and differentially expressed miRNA

**FIGURE 4:**
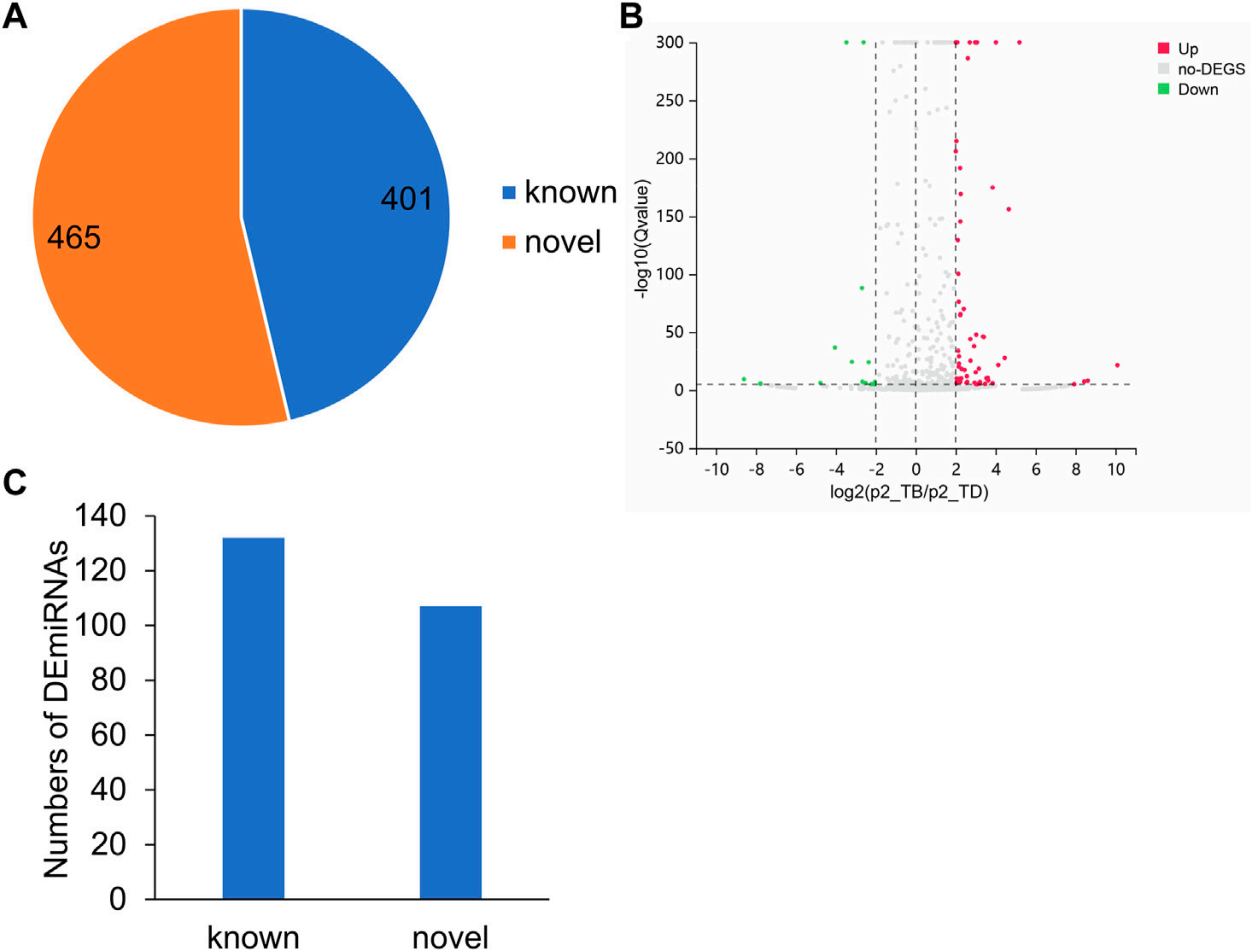
MiRNA expression patterns in the rabbit model of Perthes disease. **(A)** Number of known and novel miRNAs. **(B)** Volcano plot depicting the DEmiRNAs. The *x*-axis represents the log_2_FC and the *y*-axis represents the log_10_FDR values. The red and green dots indicate the miRNAs that were significantly upregulated and downregulated, respectively, in Perthes disease, while the gray dots indicate miRNAs that were not significantly differentially expressed. Up means upregulated, no-DEGs indicates not significant, Down indicates downregulated. **(C)** Number of known and novel significantly DEmiRNAs. The novel DEmiRNAs were identified using the miRDeep2 tool.

In order to establish a dependable ceRNA network, the samples obtained from 5 rabbits with Perthes disease and 3 control rabbits were sequenced using small RNA-seq. The miRDeep2 package was used for identifying the novel miRNAs, which led to the characterization of 465 novel miRNAs and 401 known miRNAs. The expression levels of all 866 miRNAs from the 8 samples obtained from 5 diseased rabbits and 3 healthy controls were subsequently quantified, and the DEmiRNAs were identified using the DESeq2 package in R (Figure 4A; Supplementary Table S9). The upregulated and downregulated DEmiRNAs were identified using strict screening criteria (log_2_FC > 2 or < −2, and *p*-value <0.05). A total of 189 upregulated and 50 downregulated DEmiRNAs were subsequently identified, which could have a potential role in the development of Perthes disease (Figure 4B). Notably, 132 of these DEmiRNAs were known miRNAs, while 107 were novel miRNAs (Figure 4C).

### 2.6 Identification of miRNA-target genes and lncRNAs

**FIGURE 5:**
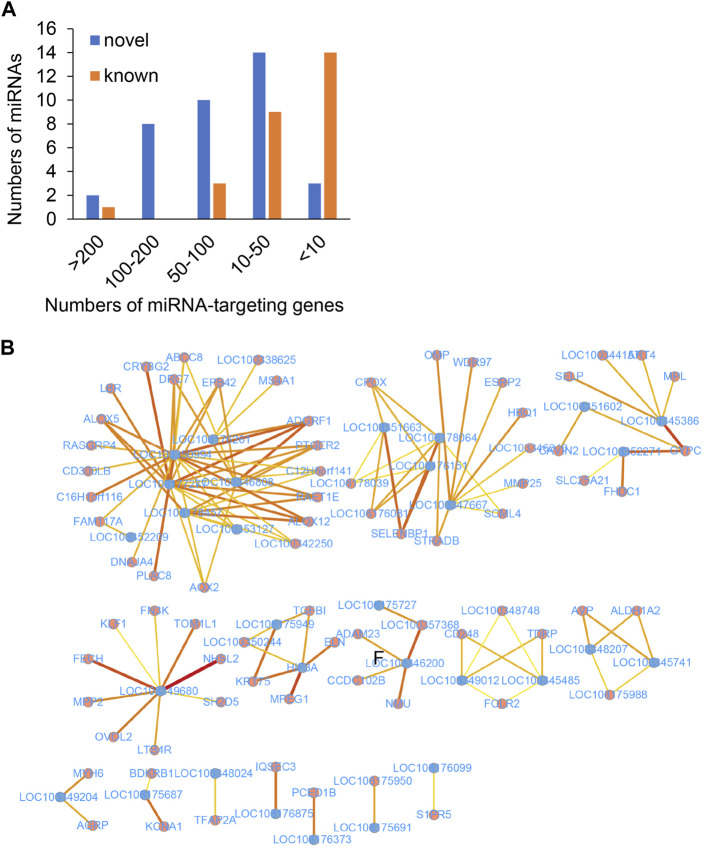
The predicted ceRNA network of key genes. **(A)** Number of target DEGs of the known (brown) and novel (blue) miRNAs. The *x*-axis represents the number of targeted DEGs, while the *y*-axis represents the number of DEmiRNAs. **(B)** The predicted ceRNA network. The blue and red dots indicate lncRNAs and mRNAs, respectively. Deeper red lines indicate the involvement of a higher number of miRNAs.

The role of lncRNAs that function as miRNA sponges and prevent the miRNA-induced degradation of mRNAs was investigated in this study. The target genes of the miRNAs were predicted using miRanda and RNAhybrid. The miRNA targets with miRanda minimum free energy (MFE) < −30, miRanda score >160, RNAhybrid MFE < −27, and RNAhybrid *p*-value <0.05 were retained for increasing the accuracy of the results. The findings revealed that the majority of DEmiRNAs targeted less than 50 genes in the genomic range (Figure 5A). Interestingly, the *ocu-miR-12093-3p* miRNA had 293 potential binding targets, suggesting that this miRNA plays a crucial role in the development of Perthes disease.

**FIGURE 6:**
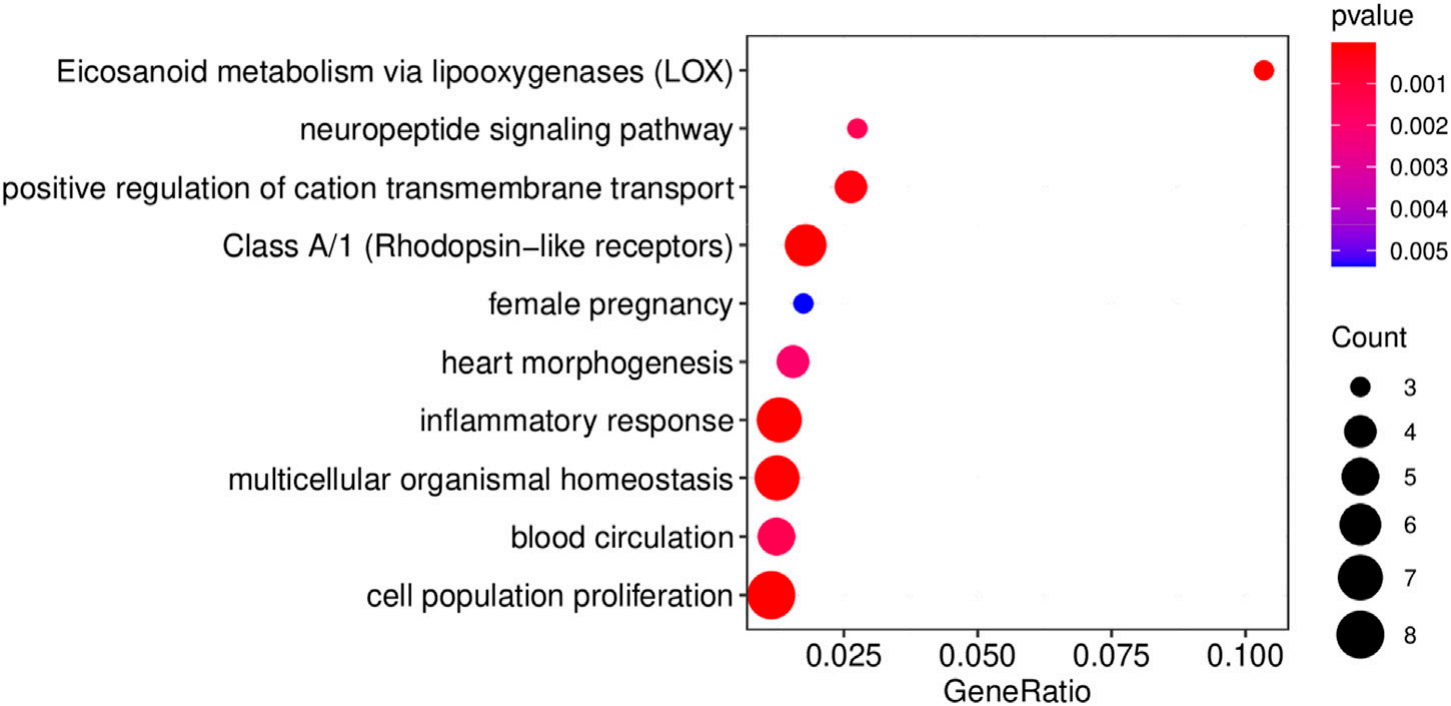
The results of functional enrichment of the hub genes in the ceRNA network. Results of GO/KEGG pathway enrichment analysis of the hub genes in the ceRNA network. Enrichment analyses were performed using MetaScape (Zhou et al., 2019).

A comprehensive lncRNA-miRNA-target gene ceRNA network was constructed by differential analysis, WGNCA clustering, and analyzing the miRNA targets (Figure 5B; Supplementary Table S10). Analysis of the ceRNA network revealed that certain hub genes, including *PTGER2* (Trau et al., 2015) and *ALOX12* (Zheng et al., 2020), were crucial for angiogenesis. Some hub genes (Figure 6), including *ALOX5, BDKRB1, LTB4R, FOLR2, PTGER2, SELP,* and *MMP25*, were related to the inflammatory response, while certain hub genes, including *AVP, BDKRB1, ELN, MYH6, ABCC8, FHDC1, KCNA1,* and *NMU,* were related to blood circulation. The inflammatory response and blood circulation play a crucial role in the development of Perthes disease (Krutikova and Vinogradova, 2015; Spasovski et al., 2023). Altogether, analysis of the ceRNA network revealed several aspects related to the development of Perthes disease, which can provide insights for further studies in this regard.

## 3 Discussion

A rabbit model of Perthes disease was developed in this study, and subsequent morphological analysis and sequencing demonstrated that this model is ideal for investigating the phenotypes, syndromes, and pathogenesis of Perthes disease. For model generation, the teres ligament of the femoral head of 8-week-old rabbits was disrupted, which disrupted the blood flow and resulted in the development of Perthes disease. As depicted in Figure 1, the morphology of the disrupted femoral head exhibited a loss of spherical structure, pale coloration, thickened cartilage layer, and poor gloss, which are commonly observed in children with Perthes disease (Catterall et al., 1982; Doria et al., 2008; Memiş et al., 2018; Shapiro, 2019). X-ray visualization of the femoral heads of the rabbit model revealed a heterogeneous bone density, localized collapse, and loss of spherical structure, which are also observed in children with Perthes disease (Madan et al., 2003; Rich and Schoenecker, 2013; Cheng et al., 2014). These observations validate that the rabbit model of Perthes disease constructed in this study was suitable for studying the mechanisms underlying the development of Perthes disease.

Perthes disease is a condition that affects the hip joint in children and occurs due to a disruption in the blood flow to the femoral head, which can result in the death of the bone tissue. Although the exact cause underlying the development of Perthes disease remains to be elucidated, previous studies suggest that a combination of genetic and environmental factors is involved in the pathogenesis of Perthes disease.

To date, only a few studies have investigated the gene expression profile in Perthes disease using microarray or miRNA-seq approaches (Huang et al., 2022; Wang et al., 2022). The present study therefore aimed to perform whole-transcriptome sequencing combined with miRNA sequencing for analyzing the gene expression patterns in Perthes disease. In order to investigate the molecular mechanisms underlying the pathogenesis of Perthes disease, the differences in gene expression between the rabbit model of Perthes disease and healthy controls were compared. A total of 1027 DEGs, 77 DElncRNAs, and 239 DEmiRNAs were identified by bioinformatics analysis.

Previous studies have demonstrated that certain biomarkers play a crucial role in the development of Perthes disease. For instance, *VEGF* plays an important role in promoting the growth and repair of blood vessels that may be disrupted in individuals with Perthes disease (Kim et al., 2004). Additionally, the occurrence of inflammation is fundamental to the pathogenesis of Perthes disease, and previous studies have reported that genetic variations in certain inflammation-related genes, including *IL-6*, *TLR4*, *TNFA*, and *IL-3*, are associated with the development of this condition (Srzentić et al., 2014; Teplen’kiy et al., 2020).

Previous studies have demonstrated that certain cytokines, including *IL-6*, *IL-1β*, and *tumor necrosis factor (TNF)-α*, are overexpressed in individuals with Perthes disease. Interestingly, the administration of tocilizumab, an inhibitor of *IL-6*, suppresses the increased expression of these cytokines in osteonecrotic articular chondrocytes during culture under hypoxic conditions (Ren et al., 2021). The findings of the present study were consistent with these reports, and revealed that the *IL-6* gene was significantly upregulated in the rabbit model of Perthes disease.

It has been reported that acute-phase response, eosinophil chemotaxis, cytokine-cytokine receptor pathways, and lysosomal pathways are involved in the pathogenesis of Perthes disease. Acute-phase response is a cellular response mechanism to injury, where inflammatory cells secrete various cytokines, including *IL-1*, *IL-6*, *TNF-α*, and *IGF-I*, among others. The acute-phase response plays an important role in collagen synthesis and growth in Perthes disease (Johannesen et al., 2009). An increase in eosinophilia indicates the occurrence of extensive necrosis. As Perthes disease develops in a sterile environment, the genes related to bacterial resistance is downregulated (Knight et al., 2008). The suppression of heme biosynthesis also plays an important role in the development of Perthes disease. Heme is an essential prosthetic group of hemoglobin and myoglobin, which have various catalytic functions, and the bone marrow is the major tissue for heme synthesis (Ogun et al., 2019).

The findings of the present study are highly consistent with the results of previous reports, which indicates that the rabbit model of Perthes disease constructed herein and the analytical approaches employed in the study could be reliably applied for determining the gene expression patterns in Perthes disease. WGCNA was performed for clustering the DEGs identified in the study. A total of seven DEG clusters were obtained by WGCNA based on the expression patterns. As depicted in Figure 4C, the DEGs in the different clusters were involved in completely different biological processes. The upregulated DEGs in group G6 (brown) were primarily enriched in the *TLR4* signaling pathway, proteolysis, *Wnt* signaling pathway, and other terms. Using a piglet model, a previous study reported that the expression of *TLR4* increases in Perthes disease (Srzentić et al., 2014), while another study reported that the rate of proteolysis is higher in Perthes disease (Eckerwall et al., 1997). Additionally, the genes in the *Wnt* signaling pathway are known to play an important role in the formation and maintenance of the cartilage-bone interface (Luyten et al., 2009). The expression levels of the DEGs in group G2 (blue) were higher than those of the control group, and primarily involved in bacterial resistance. This finding corroborates with the fact that Perthes disease develops in a sterile environment. The results of KEGG pathway enrichment analysis revealed that the DEGs in group G6 were involved in autophagy and lysosome signaling pathways, which play an important role in the pathogenesis of Perthes disease and necrosis of the femoral head (Luo et al., 2018b).

The samples obtained from the rabbit model of Perthes disease were sequenced using miRNA-seq to obtain a comprehensive gene regulatory network. To this end, the DEmiRNAs in Perthes disease were screened for constructing an lncRNA-miRNA-mRNA ceRNA network. The kinds of known miRNAs in rabbits is lower than those of humans. The miRDeep2 tool was used to predict the novel miRNAs in the sequencing data, and a cohort of known and novel miRNAs was subsequently quantified. The known and novel DEmiRNAs in Perthes disease were identified by differentially expression analysis. The target genes of the miRNAs were subsequently determined using RNAHybrid and miRand for constructing a comprehensive ceRNA regulatory network. However, further studies and experimental validation are necessary for determining the mechanism of regulation of these ceRNAs.

## 4 Materials and methods

### 4.1 Construction of animal model and confirmation of Perthes disease phenotype

Wild-type rabbits were purchased from Jinan Jinfeng Experimental Animal Company Limited, China. The rabbit model of Perthes disease was generated by incising the hip joints of 8-week-old rabbits to expose the femoral neck, which was ligated with an elastic non-absorbable suture. The ligamentum teres of the femoral head was transected to obstruct the blood flow to the epiphysis of the femoral head. The use of experimental animals was approved by the Ethics Committee of Affiliated Hospital of Zunyi Medical College (approval ID: KLL-2020-008). The experimental protocols were approved by the licensing committee of the Ethics Committee of Affiliated Hospital of Zunyi Medical College.

### 4.2 RNA isolation and quality control

The tissues of femoral head were prepared and the total RNA was extracted using TRIzol reagent (Thermo Fisher Scientific, Waltham, MA, United States) according to the manufacturer’s instructions. The extracted RNA was purified and quantified using a NanoPhotometer^®^ spectrophotometer (IMPLEN, CA, United States). The integrity of the extracted RNA was evaluated using an RNA Nano 6000 kit, with a 2100 Bioanalyzer (Agilent Technologies, CA, United States).

### 4.3 Library preparation for total RNA-seq and small RNA sequencing

A 5 μg aliquot of RNA from each sample was used for total RNA-seq, and the ribosomal RNA was removed using an Epicentre Ribo-zero™ rRNA Removal Kit (Epicentre, United States). The RNA-seq libraries were subsequently prepared using a NEBNext^®^ UltraTM Directional RNA Library Prep Kit for Illumina (NEB, United States). Sequencing was performed using an Illumina Hiseq 4000 platform to generate 150 bp paired-end reads.

For small RNA-seq, a 3 μg aliquot of RNA was obtained from each sample and processed using a NEBNext^®^ Multiplex Small RNA Library Prep Set for Illumina^®^ (NEB, Ipswich, MA, United States). The sequencing library was next prepared using TruSeq PE Cluster Kit v3-cBot-HS. Sequencing was performed using an Illumina Hiseq 2500 platform to generate 50 bp reads.

### 4.4 Quality control and read mapping

The reference genome and annotation files were retrieved from the NCBI Assembly database (accession ID: GCA_000003625.1). The adapter sequences, low-quality reads (numbers of quality lower than 15 bases being higher than 20%), and reads with more than 5% unknown bases were discarded using the SOAPnuke tool, v1.5.2 (Chen et al., 2018). The quality of the clean reads was evaluated using the fastQC tool, v0.11.8 (Andrews, 2010). The clean reads were mapped to the reference genome with HISAT2 v2.0.4 (Kim et al., 2015), using default parameters. The gene expression levels were subsequently quantified using RSEM v1.1.17 (Li and Dewey, 2011) and HTSeq v1.99.2 (Anders et al., 2015).

### 4.5 Identification of novel miRNAs

The adapter sequences in the miRNA-seq data were removed using the cutadapt tool, v4.0 (Martin, 2011), and the length distribution of the clean reads was compared. The novel miRNAs were identified using miRdeep v2.0.0.5 (Friedländer et al., 2008), and the quality of the prediction of novel miRNAs was evaluated based on the miRdeep scores and the true positive rate.

### 4.6 Identification of DEmRNAS, DElncRNAs, and DEmiRNAs

In order to identify the DElncRNAs and DEmRNAs, the DEGs were first identified from the read counts using DESeq2 v1.36.0 (Love et al., 2014). The genes with significant differential expression were identified based on the following criteria: log_2_FC > 2 or < −2 and FDR values < 1e-5. The clustering heatmaps of DEmRNAs and DElncRNAs were subsequently prepared using ComplexHeatmap v2.10.0 (Gu et al., 2016).

In order to identify the DEmiRNAs, the DEGs were initially identified based on the read counts using DESeq2 v1.36.0. The genes with significant differential expression were identified based on the following criteria: log_2_FC > 1 or < −1 and FDR values < 1e-5. The DEmiRNAs were represented by volcano plots prepared using the ggplot2 package (v3.3.6) in R 4.1.0 (Villanueva and Chen, 2019).

### 4.7 GO and KEGG pathway enrichment analyses

The DEmRNAs were subjected to GO (Consortium, 2019) and KEGG pathway (Kanehisa and Goto, 2000) enrichment analyses for determining their biological functions. The DEmRNAs were separated into two groups, namely, the upregulated (log_2_FC > 2) and downregulated (log_2_FC < −2) groups, based on the log_2_FC values. The results of GO and KEGG pathway enrichment analyses of the two groups were analyzed using the clusterProfiler package (v4.2.2) in R (Yu et al., 2012). Terms with *p*-values <0.05 were considered to be significantly enriched.

### 4.8 Construction of WGCNA network

The DEGs were identified using the WGCNA package (v1.70.3) in R (Langfelder and Horvath, 2008) for constructing the WGCNA network. Modules with module significance <0.05 were identified as recurrence-associated modules.

The clustered genes identified by WGNCA were subjected to GO and KEGG pathway enrichment analyses using the clusterProfiler package (v4.2.2) in R for predicting their biological functions (Yu et al., 2012). Terms with *p*-value <0.05 were considered to be significantly enriched.

### 4.9 Prediction of lncRNA and mRNA targets of miRNAs

The mRNA and lncRNA targets of the miRNAs were predicted using the RNAhybrid v2.1.2 (Krüger and Rehmsmeier, 2006) and miRanda v3.3a (Betel et al., 2010) tools, with default parameters. In order to improve the accuracy of the prediction, targets with miRanda scores >160, MFE < −30, RNAhybrid MFE < −20, and *p*-value <0.05 were retained.

### 4.10 Construction of ceRNA network

According to the ceRNA hypothesis, the conversely regulated DElncRNA and mRNA pairs were used for constructing the ceRNA network in this study. The important DEGs were selected based on the results of miRNA target prediction for constructing the lncRNA-miRNA-mRNA ceRNA network, which was visualized using Cytoscape v2.9 (Shannon et al., 2003).

## Data Availability

The data presented in the study are deposited in the NCBI Gene Expression Omnibus repository, accession number GSE217908.
